# Minimalist Footwear Acutely Alters Running Kinematics in Runners With Medial Tibial Stress Syndrome

**DOI:** 10.1002/ars2.70005

**Published:** 2026-04-30

**Authors:** Quyet Thang Nguyen, My T. Than, Minh Ho Ngoc, Ngoc‐Tram Nguyen, Nam Vu Tu, Tien Quang Tran, Dung Tran Trung

**Affiliations:** ^1^ Center for Orthopaedics and Sports Medicine Vinmec Times City International Hospital Hanoi Vietnam; ^2^ College of Health Science VinUniversity Hanoi Vietnam

## Abstract

**Purpose:**

To assess immediate biomechanical changes in runners with medial tibial stress syndrome (MTSS) when transitioning from standard to minimalist running shoes.

**Methods:**

Active runners diagnosed with MTSS ran on a treadmill at 10 km/h using standard and minimalist running shoes. Hip, knee, and ankle joint kinematics were captured using a 16‐infrared‐camera Vicon motion capture system. Strike patterns were estimated from the foot strike angle. Comparison between footwear types was performed using Wilcoxon signed‐rank tests for continuous variables and Stuart‐Maxwell tests for categorical variables.

**Results:**

In total, 25 runners (19 male, 6 female, 30.1 ± 6.4 years old) participated in data collection throughout June 2025. Footwear change resulted in a significant decrease in peak stance‐phase flexion of left (1.7° ± 4.7°, *P * = .040) and right (2.7° ± 6.6°, *P* = .024) knees, decreased stride length (2.9° ± 3.6 cm, *P * < .001), and increased cadence (2.7 ± 3.4 steps/minute, *P*  < .001). No significant alteration was observed for peak stance‐phase hip internal rotation, frontal‐plane pelvic tilt, and foot strike patterns.

**Conclusions:**

Although changes in stride length and cadence were favorable, decreased knee flexion might compromise the knee shock‐absorbing function of minimalist running shoes. Absence of change in strike patterns and proximal joint kinematics indicated that short‐term footwear modification alone might be insufficient to alter running kinematics in patients with MTSS.

**Clinical Relevance:**

MTSS, also referred to as shin splints, is a common overuse injury. Adjusting running biomechanics by selecting appropriate footwear has been recommended for injury prevention; however, the best type of footwear remains unknown for this population. The results of this study may help inform evidence‐based recommendations and strategic interventions to improve MTSS outcomes in affected runners.

## Graphical abstract

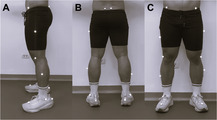

Medial tibial stress syndrome (MTSS), also referred to as shin splints, is an overuse injury commonly occurring among athletes and military recruits with incidence varying from 4% to 35% in these populations.^1^ Lopes et al. identified MTSS as the main running‐related musculoskeletal injury (incidence ranging from 13.6% to 20.0% and prevalence of 9.5%).^2^

Numerous studies suggested that biomechanical factors such as ground reaction force, knee flexion (KF), hip movement, cadence, and foot strike pattern (FSP) were strongly associated with MTSS.^3^, ^4^, ^5^, ^6^ Loudon et al. found that subjects with a history of MTSS displayed significantly greater frontal‐plane pelvic tilt (PT) and peak hip internal rotation (HIR) and reduced knee flexion compared with the control group.^7^ Meanwhile, the study by Kuwabara et al. study associated lower cadence with a 6.7‐fold increase in the likelihood of shin injury among high school runners.^8^ Moreover, Daoud et al. observed that runners who rearfoot strike (RFS) had more than twice the likelihood of developing repetitive stress injuries, including MTSS, compared with forefoot strikers.^3^

Adjusting running biomechanics by selecting appropriate footwear has been recommended for injury prevention.^9^ Athletes are advised to select shoes with adequate shock absorption to reduce forces transmitted through the lower limbs and prevent recurrent episodes of MTSS.^6^ However, the optimal running footwear remains debated, with no conclusive evidence supporting a specific recommendation.^8^, ^10^ The benefits of a particular footwear type for individuals with MTSS remain inconclusive. Moreover, research investigating the effects of different footwear types on running kinematics in individuals with MTSS is limited. Most existing studies focus on healthy populations, leaving a notable gap in biomechanics literature regarding those affected by this condition.^11^, ^12^

The purpose of this study was to assess immediate biomechanical changes in runners with MTSS when transitioning from standard to minimalist running shoes (MRS). We hypothesized that minimalist footwear transition would lead to acute alterations in key running kinematics, including a shift from rearfoot strike to forefoot strike (FFS)/midfoot strike (MFS), increased KF, decreased PT and HIR, higher cadence, and shorter stride length during running.

## METHODS

### Study Design and Participants

This was a cross‐sectional, repeated‐measures study assessing kinematic and spatiotemporal variables among runners with MTSS under different footwear conditions. Participants in this study were recruited online via social media platforms, using announcements written in Vietnamese, the local language. These posts targeted recreational runners and were distributed through community groups and forums specific to running and fitness.

Participants were included if they were aged between 18 and 60 years; were active runners who trained minimum distance of 20 km/week; were diagnosed with acute or chronic uni‐/bilateral MTSS; had reported pain level during running of less than 5/10 on Visual Analog Scale; experienced reduction in running with no ongoing medical treatments for their condition; and had no experience using MRS to ensure no previous biomechanical adaptation.

Exclusion criteria were history of lower limb stress fractures, compartment syndrome, or distal nerve issues; any trauma or surgery involving lower extremities within the past 12 months; known knee conditions or history of knee surgery; current lower back pain or chronic hip or knee pain; paresthesia in lower limbs; structural abnormalities of lower extremities (e.g., pes planus, excessive foot pronation determined by navicular drop test, genu valgum/varum); known soft tissue or tendon disorders in lower extremities; or presence of osteoarthritis or other degenerative joint diseases.

In this study, MTSS was diagnosed based on a combination of clinical criteria and supporting magnetic resonance imaging findings, aligned with widely accepted frameworks, such as one described by Yates and White.^13^ MTSS was defined as dull, aching pain along the distal two‐thirds of the posteromedial tibial border, aggravated by running and relieved with rest. Tenderness palpation was diffuse and extended over 5 cm along the tibial border.^13^ Diagnosis was further confirmed with magnetic resonance imaging findings: periosteal edema along the posteromedial tibial cortex without cortical disruption or bone marrow. Diagnosis confirmation was done by 2 investigators, a licensed physiotherapist (Q.T.N.) and an orthopaedic surgeon (M.H.N.).

The sample size of 25 was estimated by conducting a priori power analysis following guidelines by Sinclair et al.^14^ Sinclair's investigation closely aligned with the current study's objectives. Significance level of 5% (*α* = 0.05) and statistical power of 80% (1‐*β* = 0.80) were chosen to ensure adequate sensitivity in detecting meaningful kinematic differences.

Informed consents from all participants and institutional ethical approval were obtained before the commencement of data collection.

### Footwear Types

In this study, standard running shoes (SRS) refers to regular cushioned running footwear with heel‐to‐forefoot drop exceeding 10 mm.^15^ MRS refers to running footwear adopting the minimal design that has <7‐mm heel‐to‐forefoot drop, <200‐g weight, and little to no cushioning, as defined by Coetzee et al.^16^

### Subject Preparation

The day prior to testing, participants were instructed not to engage in intense physical activities, workouts, and exercise. Caffeine consumption was avoided 12 hours before testing.

Participants’ demographic and anthropometric data were recorded. Anthropometric measurements and marker placement were performed by the same physiotherapist throughout the study.

**FIGURE 1:**
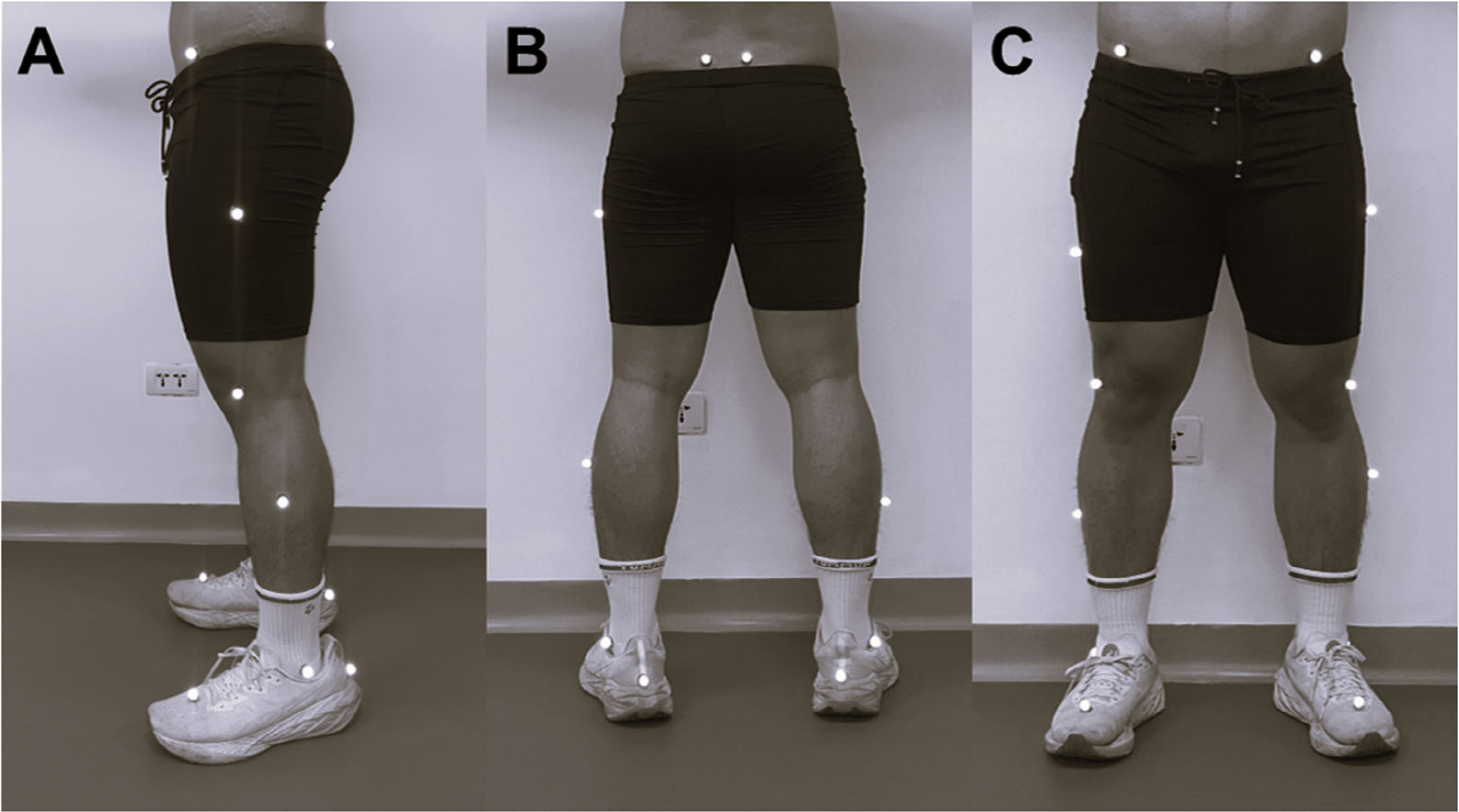
Marker attachment following the Vicon Plug‐in Gait Lower Body model: (A) lateral, (B) posterior, and (C) frontal views.

Sixteen 14‐mm‐diameter reflective markers (B&L Engineering, Santa Ana, CA, USA) were placed bilaterally on key anatomical landmarks of the lower limbs of each participant to construct the skeletal model for motion capturing. These markers are attached using double‐sided tape onto the following sites: anterior superior iliac spine, posterior superior iliac spine, lateral thigh surface, lateral femoral epicondyle (knee marker), lateral shank surface, lateral malleolus (ankle marker), second metatarsal head (toe marker), and posterior calcaneus (heel marker). Marker attachments were based on the Plug‐in Gait Lower Body model (Vicon Motion Systems Ltd, Oxfordshire, UK) (Figure 1).

Before running trials, each participant underwent standing calibration trial to establish segment lengths, joint centers, and joint coordinate systems. All participants engaged in a warm‐up routine in their selected SRS, incorporating full‐body dynamic stretching, followed by a 5‐min walk at 4.8km/h and an additional 3‐5 min of jogging.

### Running Trials

Running trials were conducted on a treadmill (Quasar Med h/p/cosmos 170‐190/65, H/p/cosmos Sports & Medical GmbH, Nussdorf‐Traunstein, Germany) with an integrated plantar pressure sensor (FDM‐THQ system, Zebris Medical GmbH, Baden‐Württemberg, Germany) under 2 footwear conditions: SRS and MRS. Under the SRS condition, participants wore their own running shoes. Heel‐to‐forefoot drop was noted for each shoe model. Under the MRS condition, participants wore a generic‐brand MRS that had a 2‐mm heel‐to‐forefoot drop.

Participants were required to sustain 10‐km/h running speed as established by prior studies.^12^, ^17^ With each type of footwear, participant's performance was recorded in 3 separate trials, each lasting 10 s, and data were collected at the 2.5‐min point during the 10‐km/h run.^7^, ^18^

No adaptation period was allowed for transitioning from SRS to MRS, as this study emphasized the immediate effects of switching footwear on running mechanics.

### Data Capture

Kinematic data of pelvis, hip, knee, and ankle during treadmill running were captured by 16 high‐speed 3D infrared cameras (Valkyrie VK26, Vicon Motion Systems Ltd, Oxfordshire, UK) at 250‐Hz sampling frequency. A laboratory‐based coordinate system was consistently used throughout this study, with the positive x‐axis oriented anteriorly relative to the participant, the positive y‐axis positioned to the participant's left, and the positive z‐axis aligned superiorly relative to the participant.

Captured data were processed and analyzed using Vicon Nexus 2.16 software (Vicon Motion Systems, Oxfordshire, UK). Kinematic variables of interest were computed using a Python script. Stride length and cadence were determined from plantar pressure data using MR4 software (Noraxon USA, Scottsdale, AZ, USA).

### Outcome Measures

Outcome variables of interest included FSP, KF, and HIR peak angles during the stance phase, PT, stride length, and cadence.

FSP was estimated from the foot strike angle as established by Altman and Davis and used in other studies.^19^, ^20^, ^21^ Foot strike angle was determined by subtracting the ankle angle at static standing from the ankle angle at initial contact. Ankle angle was measured between the foot vector (extending from the heel to toe markers) and the sagittal axis of the shank, with a positive value corresponding to dorsiflexion. Following Altman and Davis, RFS, MFS, and FFS, respectively, corresponded to strike angle ranges of >8.0°, −1.6° to 8.0°, and <−1.6°.

KF was defined as the angle between the femur and tibia. HIR was the angle between the femur and pelvis along the vertical axis. PT was the angle formed between the ASIS line and the laboratory‐defined horizontal reference.

### Statistical Analysis

Descriptive statistics were calculated for demographic data and all kinematic variables. Quantitative data were reported as mean ± standard deviation unless noted otherwise. Shapiro‐Wilk tests were employed to check the normality of each examined variable.

Wilcoxon signed‐rank tests were used to compare kinematic variables, cadence, and stride length when running with SRS with those when running with MRS. Comparison of kinematics and kinematic changes due to footwear transition was done between the left and right legs. Stuart‐Maxwell tests were used to compare FSP. In addition to aggregated data analysis, exploratory sex‐disaggregated analysis was also performed using the same tests with results presented in the Supporting Information.

Statistical analysis was completed using IBM SPSS 29.0 software (IBM SPSS Statistics, Armonk, NY, USA). Significance level was set to *P * = .05 for all tests.

## RESULTS

### Participant Characteristics

In total, 25 active runners (19 male, 6 female) running 35 ± 10 km/week participated in data collection throughout June 2025. All reported to have experienced treadmill running before. Participants’ demographic characteristics and anthropometric data are shown in Table 1. Mean age was 30.1 ± 6.4 years (range: 20‐45). Average body mass index was 23.2 ± 2.2. Participants wore SRS with a mean heel‐to‐forefoot drop of 11.9 ± 1.7 mm, aligning with typical design specifications. Affected limb distribution was 28% left, 44% right, and 28% bilateral.

**TABLE 1:** Demographic and Anthropometric Data of the Study Subjects and the Average Heel‐to‐Forefoot Drops of Their Own Standard Running Shoes Used During Normal Running Condition

**Parameter**	**Aggregated (N = 25)**	**Male (N = 19 [76%])**	**Female (N = 6 [24%])**
**Age [range], yr**	30.1 ± 6.4 [20‐45]	30.1 ± 6.5 [20‐45]	30.0 ± 6.9 [24‐42]
**Affected limb**:			
‐ Left	7 (28%)	5 (26%)	2 (33%)
‐ Right	11 (44%)	9 (47%)	2 (33%)
‐ Bilateral	7 (28%)	5 (26%)	2 (33%)
**Ankle width, cm**	7.0 ± 0.5	7.0 ± 0.3	6.8 ± 0.9
**Knee width, cm**	8.8 ± 0.7	8.7 ± 0.6	9.3 ± 0.8
**Leg length, cm**	86.2 ± 5.4	87.7 ± 5.1	81.5 ± 2.9
**Height, cm**	167.7 ± 8.3	170.8 ± 6.9	158.1 ± 3.7
**Weight, kg**	65.3 ± 8.4	67.8 ± 7.7	57.2 ± 5.1
**BMI**	23.2 ± 2.2	23.3 ± 2.4	22.9 ± 1.8
**Heel drop, mm**	11.9 ± 1.7	12.3 ± 1.7	10.3 ± 0.4

*Note*: Data are displayed as mean ± standard deviation unless noted otherwise.

BMI, body mass index; cm, centimeters; kg, kilograms; mm, millimeters; yr, year.

### Aggregated Kinematics Comparison Between SRS and MRS

Shapiro‐Wilk tests were nonsignificant for all variables except left and right PT under SRS condition and right PT under MRS condition.

**FIGURE 2:**
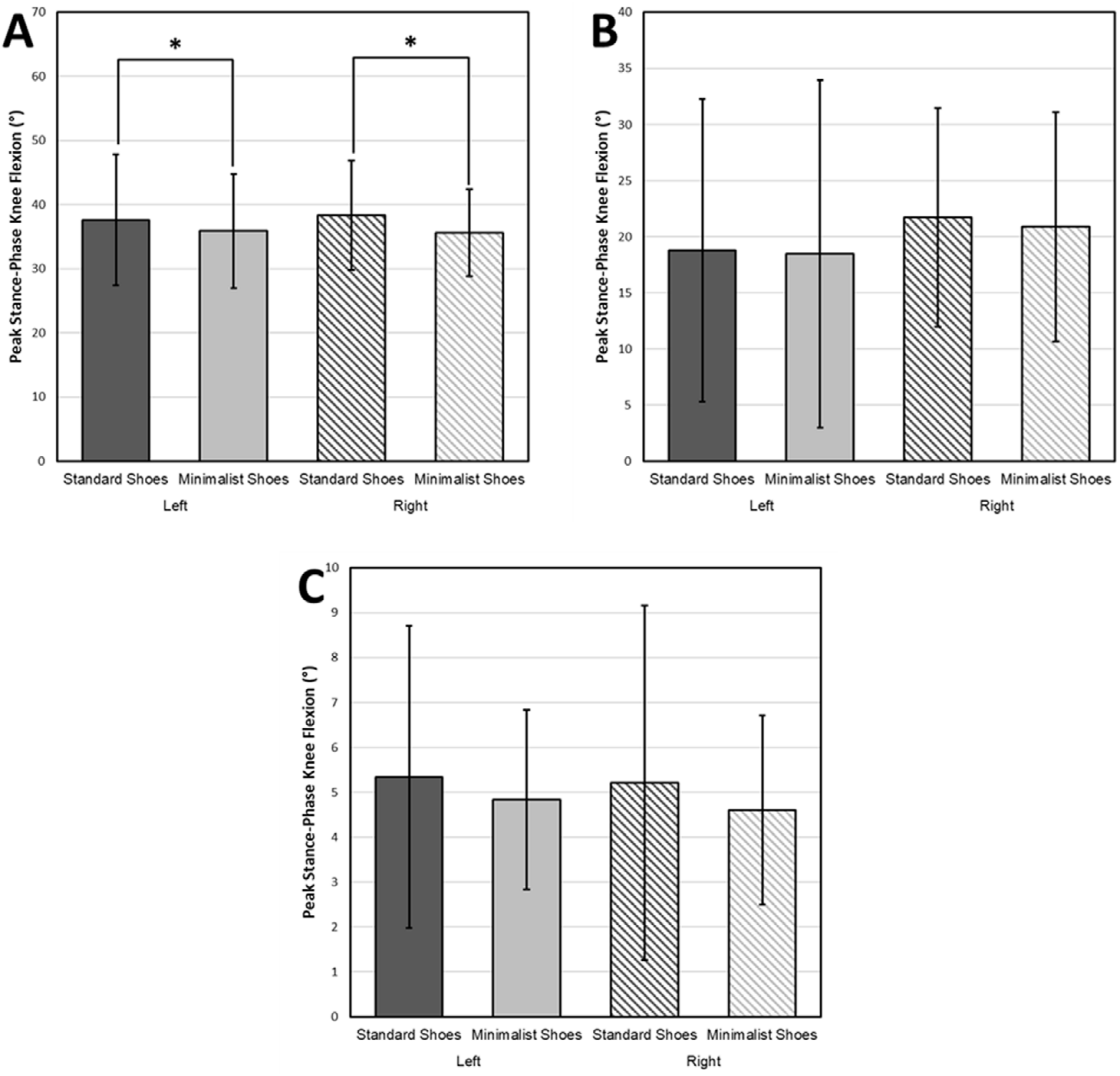
Kinematic variables when running with standard running shoes and minimalist running shoes: (A) peak knee flexion angle during the stance phase, (B) peak hip internal rotation angle during the stance phase, and (C) pelvic tilt in the frontal plane. *Significantly different with *P *< .05.

Peak stance‐phase KF significantly decreased by 1.7° ±4.7° (*P *= .012) for the left knee and 2.7° ± 6.6° (*P *= .020) for the right knee after switching from SRS to MRS (Table 2, Figure 2A).

**TABLE 2:** Average Kinematic Variables When Wearing Standard Running Shoes and Those When Wearing Minimalist Running Shoes and Average Kinematic Changes After Transition to Minimalist Running Shoes

**Kinematic Variable**	**Laterality**	**SRS**	**MRS**	**Change**	***P*** **Value**
**Peak knee flexion during stance phase, °**	Left	37.6 ± 10.2	35.9 ± 8.9	−1.7 ± 4.7	**.012** *
	Right	38.4 ± 8.5	35.6 ± 6.8	−2.7 ± 6.6	**.020** *
**Peak hip internal rotation during stance phase, °**	Left	19.2 ± 13.8	18.5 ± 15.5	−0.7 ± 7.0	.510
	Right	21.7 ± 9.8	20.9 ± 10.2	−0.8 ± 7.0	.397
**Frontal plane pelvic tilt, °**	Left	5.3 ± 3.4	4.8 ± 2.0	−0.5 ± 3.6	.882
	Right	5.2 ± 3.9	4.6 ± 2.1	−0.6 ± 2.9	.581
**Stride length, cm**	‐	191.8 ± 15.8	188.8 ± 14.7	−2.9 ± 3.6	**.001** *
**Cadence, step/min**	‐	174.7 ± 14.0	177.4 ± 13.9	2.7 ± 3.4	**.002** *

*Note*: *P* values were from Wilcoxon signed‐rank tests.

MRS, minimalist running shoes; SRS, standard running shoes.

* Significantly different between kinematic variables under 2 footwear conditions with *P *< .05.

There was no significant difference in peak stance‐phase HIR and PT between running with SRS and MRS (Table 2, Figure 2B,C).

**FIGURE 3:**
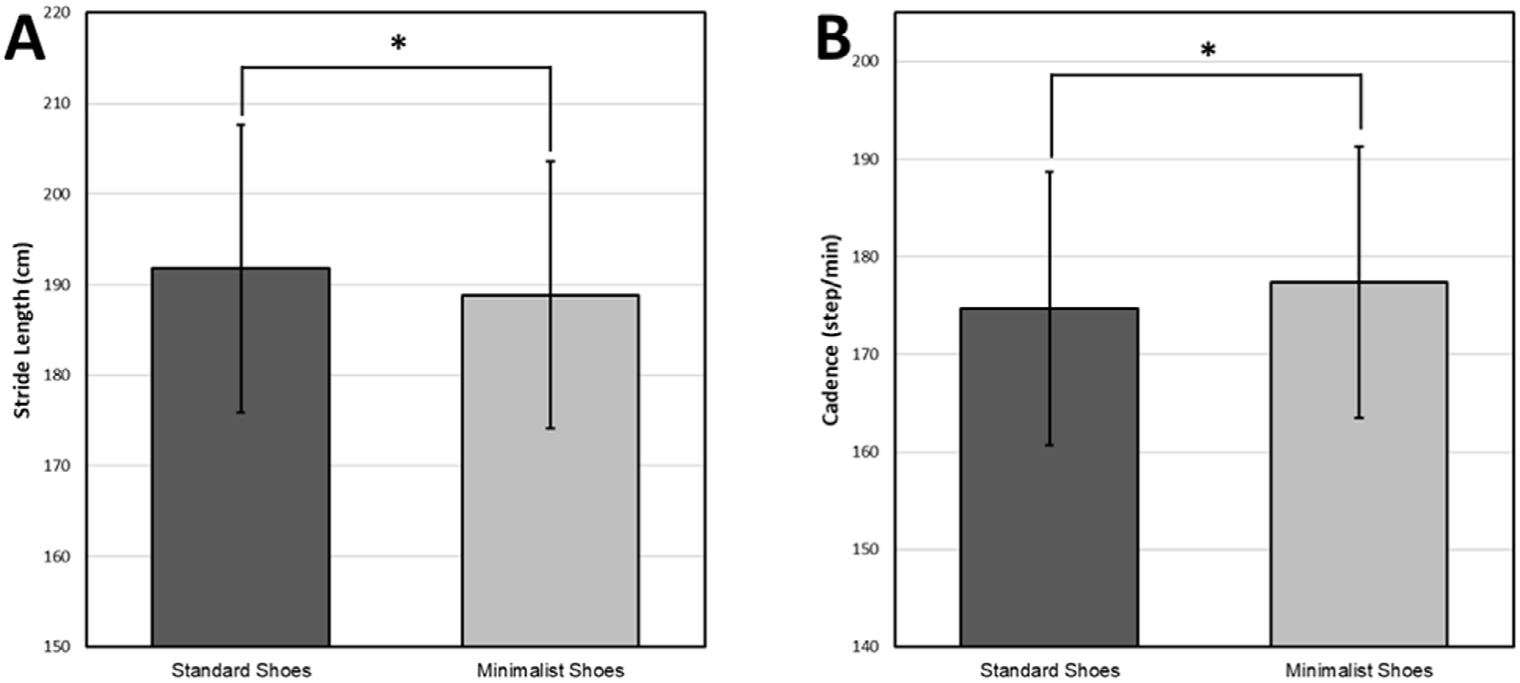
Stride length (A) and cadence (B) when running with standard running shoes and minimalist running shoes. *Significantly different with *P *< .05.

Switching to MRS caused a significant decrease in stride length by 2.9 ± 3.6 cm (*P *= .001) and a significant increase in cadence by 2.7 ± 3.4 steps/minute (*P* = .002) (Table 2, Figure 3).

There is no significant difference between left and right legs regarding kinematic variables and kinematic changes due to footwear transition.

Stuart‐Maxwell tests detected no significant change in FSP when switching to MRS, as can be seen from Table 3.

**TABLE 3:** Frequencies of Different Foot Strike Patterns in Each Foot When Running Under Different Footwear Conditions

**Foot Laterality**	**Strike Pattern**	**SRS**	**MRS**	***P*** **Value**
**Left**	FFS	13 (52%)	8 (32%)	.157
	MFS	9 (36%)	14 (56%)	
	RFS	3 (12%)	3 (12%)	
**Right**	FFS	13 (52%)	9 (36%)	.249
	MFS	8 (32%)	12 (48%)	
	RFS	4 (16%)	4 (16%)	

*Note*: Data are presented as frequency (percentage). *P*‐values were from Stuart‐Maxwell tests.

FFS, forefoot strike; MFS, midfoot strike; MRS, minimalist running shoes; RFS, rearfoot strike; SRS, standard running shoes.

### Sex‐Specific Kinematic Comparison Between SRS and MRS

Sex‐disaggregated analysis results were presented in the Supporting Information.

For male participants, significant changes were observed for peak stance‐phase KF (both left [*P *= .012] and right [*P *= .020] knees), stride length (*P *= .001), and cadence (*P *= .002), whereas no significance was observed for peak HIR during stance, PT, and FSP (Table S1 and S2).

Meanwhile, for female participants, a significant decrease was detected for peak HIR during stance of the right hip, whereas no significant difference was observed in peak stance‐phase KF, left peak HIR, PT, stride length, cadence, and FSP (Table S1 and S2).

## DISCUSSION

Assessing the kinematics of MTSS runners wearing different footwear revealed that immediate transition to MRS significantly reduced peak stance‐phased KF, increased cadence, and decreased stride length while leaving hip kinematics and FSP unchanged. Our findings support and expand on previous literature on MRS, offering possible explanations for conflicting results in earlier studies.

### Foot Strike Pattern

Many studies agreed that highly cushioned shoes encourage RFT, whereas minimalist shoes encourage MFS and FFS patterns.^5^, ^22^, ^23^ RFS generates an additional conspicuous impact peak right after initial contact, which is unnoticeable in FFS and MFS, meaning loading rate and magnitude of RFT during the early stance phase are higher, possibly putting rearfoot strikers at higher risk of overuse injury.^3^, ^23^ Therefore, switching to minimalist footwear might be beneficial in reducing MTSS risks as they promote MFS/FFS.

In this study, however, no significant FSP alteration was observed after participants’ immediate footwear transition, differing from our initial hypothesis that switching footwear would encourage FFS.

This finding does align with other studies, which suggest FSP does not automatically change with footwear modification alone. McCallion et al. and Soares et al. found that most male recreational runners remained MFS regardless of whether they were wearing SRS, MRS, or no footwear.^24^, ^25^ Similarly, Willy et al. emphasized that MRS did not inherently induce FFS pattern, especially in the short term.^26^ Earlier studies reported that 40%‐50% of runners continued to RFS even when running barefoot or with MRS, particularly during the initial adaptation phase.^27^, ^28^ Running speed can also influence FSP: higher velocities tend to increase FFS prevalence, whereas slower endurance paces are associated with RFS.^27^, ^29^ The running speed of 10 km/h in this study can be considered moderate, sustainable pace.^30^ Interestingly, specific design characteristics of MRS also modulate the degree of gait adaptation. Squadrone et al. showed differential biomechanical effects across various MRS models.^31^

In runners with MTSS, avoiding sudden transition to FFS may serve as a compensatory mechanism to limit excessive activation of lower‐leg musculature, thereby reducing the risk of aggravating symptoms associated with the condition.^32^ Noh et al. revealed that FFS running, particularly when barefoot, resulted in significantly greater activation of the soleus and peroneal muscles in patients with MTSS compared to healthy controls, most notably, a ~21% increase in soleus activation during barefoot FFS running (*P* < .01).^32^ In contrast, no significant difference in muscle activity was observed between groups during RFS with shod conditions. These findings suggest that MTSS runners may require more muscular effort to maintain FFS, possibly due to pain or impaired neuromuscular control, thereby increasing the risk of overload if transitioning too rapidly.

Our results indicate that although MRS may facilitate subtle changes in lower‐limb biomechanics, it does not uniformly alter FSP, especially in individuals with MTSS. The observed resistance to adopting FFS may reflect protective adaptation. However, long‐term consequences of such adaptations remain unclear and warrant further longitudinal investigation to determine their implications for MTSS rehabilitation.

### Knee and Hip Kinematics

Significant reductions in peak stance‐phase KF were observed for left (−1.7°, *P *= .040) and right knees (−2.7°, *P *= .024). These findings contrast with Willy et al. and Ohmi et al., who found that MRS generally increases KF and alters running gait in a way that reduces knee loading but may increase sudden loading and foot pronation compared with traditional footwear.^26^, ^33^ One possible explanation for this discrepancy lies in differences between study populations. Previous studies mainly involved healthy male participants, whereas the current study included both male and female participants affected by MTSS. This suggests that individuals with MTSS may respond differently to changes in footwear compared with healthy individuals. However, our findings are consistent with Squadrone et al., who observed reductions in KF and altered gait patterns following short‐term exposure to MRS.^12^, ^31^ Similarly, Soares et al. reported that even experienced marathon runners exhibited noticeable gait modifications after a brief treadmill session in MRS, including reduced knee and hip flexion and shorter stride length.^25^

From a biomechanical perspective, reduced KF observed may reflect a neuromuscular compensation strategy. This response could serve to protect the tibial region from abrupt mechanical changes during ground contact after switching to MRS. Previous research indicated that MTSS was caused by repetitive mechanical loading on the medial surface of the tibia, especially due to torsional forces and bending moments during running.^34^ Increased KF during the loading phase elevates the bending moment on the tibial shaft. Additionally, the combination of KF, HIR, and foot pronation can contribute to axial torsion along the tibia, a mechanical factor implicated in MTSS pathophysiology.^35^ Therefore, reducing KF may act as a protective mechanism by limiting tibial oscillation and reducing periosteal stress.

MRS has been shown to increase runners' perceived ground stiffness. In response, runners may adapt by straightening their legs at initial contact to enhance proprioceptive feedback and reduce instability.^36^ In addition, Squadrone et al. reported that longer ground contact time and a tendency to adopt MFS in SRS may increase KF compared with barefoot/minimalist running.^31^

This change in landing mechanics is important in individuals with MTSS, where excessive tibial loading plays a central role in the progression of the condition.^35^ Our findings contrast with the original hypothesis that MRS would promote FFT and increase KF to enhance shock absorption.

Previous evaluation of hip kinematics during treadmill running showed that individuals with a history of MTSS and tibial stress fractures exhibited significantly greater HIR and PT compared with healthy runners.^7^, ^37^, ^38^ Increased HIR was shown to significantly raise MTSS risk, especially in female athletes, while higher PT may further exacerbate this risk by affecting lower limb alignment and loading patterns.^37^, ^39^

No statistically significant differences were observed in HIR and PT during the transition from SRS to MRS, aligning with Bonacci et al. and Zhang et al., who also reported minimal or no acute changes in pelvic and hip kinematics following a switch to MRS.^36^, ^40^ Most studies suggested that acute exposure to MRS did not lead to significant changes in HIR. Instead, noticeable adaptations were typically observed at the ankle and knee joints.^36^, ^40^ This can be attributed to the biomechanical characteristics of proximal joints, which are generally more stable and less sensitive to immediate changes in external factors such as footwear, particularly during acute interventions. This trend appears to occur in both healthy individuals and those with MTSS. Additionally, movement patterns in the hip‐pelvic region are predominantly regulated by central neuromuscular control strategies, which often require prolonged retraining before measurable biomechanical changes can take place.

### Stride Length and Cadence Adjustments

Transitioning to MRS resulted in notable biomechanical adaptations, most prominently a significant increase in cadence (+2.7 steps/min, *P *< .001). This finding aligns with previous research reporting similar increases in step frequency following changes in footwear and suggests that individuals with MTSS exhibit cadence responses comparable to those observed in healthy runners.^31^, ^41^ Subelite and trained athletes show shorter strides and higher cadence when transitioning from SRS to MRS and barefoot running.^12^, ^17^, ^36^ This modification is considered a natural strategy to reduce heel impact and improve comfort.^31^

Cadence modulation is also recognized as a key factor in neuromuscular adaptation. Ohmi et al. reported that increased cadence enhanced motor control and improved muscle activation, particularly in calf and periarticular stabilizers of the knee and ankle.^33^ These neuromuscular adjustments support more effective shock attenuation and reduce tibial bending moments, which may, in turn, lower the mechanical triggers of MTSS.^42^, ^43^, ^44^

A higher step rate has been extensively associated with injury prevention and gait retraining. By decreasing heel strike distance, peak vertical ground reaction force, and hip adduction at initial contact, cadence increases contribute to reducing tibial stress fracture risk.^45^ Musculoskeletal simulations suggested that shortening stride length could reduce tibial stress by 3%‐6%.^46^ Moreover, incremental increases in cadence (typically 5%‐10%) were shown to significantly decrease tibial loading, with 1 study showing that a 7% cadence increase reduced peak ground reaction force by 5.6%.^47^ Despite a modest ~2% increase in cadence, such changes may help reduce tibial load over time and promote beneficial neuromuscular adaptations in runners with MTSS.

### Recommendations

Although changes in cadence and stride length associated with MRS are beneficial, the lack of significant changes in FSP and hip kinematics, along with a notable reduction in KF, raises concerns. Research has indicated that maintaining RFS while wearing MRS may result in elevated vertical loading rates.^15^ As MRS offers reduced cushioning and alters foot‐ground interaction, continuing to RFS may negate potential benefits and increase reinjury risk. Conversely, FFS is commonly associated with lower tibial impact loading and may confer protective effects for runners with MTSS. However, the transition to FFS places increased mechanical demand on the triceps surae and associated tendons. In cases where MTSS involves traction‐related pain due to soleus activation, this redistribution of load may exacerbate symptoms if not properly managed. Therefore, runners are advised not to adopt FFS abruptly but instead to undergo a progressive adaptation period to allow for neuromuscular and structural acclimatization.^41^ Akin to adopting new strike patterns, minimalist footwear transition should also happen gradually, starting by walking/short‐duration running with weekly increments in MRS usage, as recommended by the protocol by Vincent et al.^48^ Evidence supported the efficacy of progressive training with MRS as McCarthy et al. observed more FFS patterns in female runners after 12 weeks using MRS compared with the control cohort.^49^

In summary, although MRS may offer biomechanical benefits for individuals with MTSS, successful clinical outcomes depend on adherence to structured, progressive training programs. Sudden changes in footwear or running technique without adequate adaptation time may increase injury risk, underscoring the importance of gradual implementation and individualized monitoring.

### Sex‐Specific Kinematic Changes

There was a large disparity in the number of male and female individuals within the research population (76% and 24%, respectively). Exploratory sex‐disaggregated analysis revealed similar significant variables (peak stance KF, stride length, and cadence) between aggregated and male‐only data, whereas female‐only data showed a different significant variable (right peak stance HIR).

Exploratory analysis was not specified in our hypothesis. Results were included in the Supporting Information and strictly used for descriptive purposes only. The female sample size (n = 6) was too small to yield statistically robust. With multiple comparisons between subgroups, the risk of type I error and potential false positives will greatly increase, even in the male group. Significant results, therefore, should be interpreted with great caution.

### Limitations

This study has limitations inherent in cross‐sectional study design. Emphasis was put on acute kinematic responses to footwear transition, and it was unable to observe long‐term kinematic adaptations. There was a large sex‐based disparity in the research population, possibly attributed to sex‐based disparity in sport participation where recruitment occurred as recruitment was rolling until the desired sample size was achieved. Exploratory results regarding the effect of sex on kinematic changes were provided for reference and should not be overinterpreted. The study lacks variation in running speeds, running surfaces, and types of minimalist shoes. For consistency and the fact that participants had to run in unfamiliar footwear, only 1 moderate running speed of 10 km/h was selected for the experiments to minimize injury risk for runners. The running environment was also limited to 1 controlled option: treadmill running with no inclination and unchanged surface for the same reason. Biomechanical variation due to running speed, surface, inclination, and other factors was outside the scope of this study, which highlighted only the acute effects of sudden footwear switching.

## CONCLUSIONS

Although changes in stride length and cadence were favorable, decreased KF might compromise the knee shock‐absorbing function of minimalist running shoes. Absence of change in strike patterns and proximal joint kinematics indicated that short‐term footwear modification alone might be insufficient to alter running kinematics in patients with MTSS.

## SUPPORTING INFORMATION

Additional supporting information can be found online in the Supporting Information section.

## DISCLOSURES

The authors (Q.T.N., M.T.T., M.H.N., N‐T.N., N.V.T., T.Q.T., D.T.T.) declare that they have no known competing financial interests or personal relationships that could have appeared to influence the work reported in this article.

## Supporting information

Supplementary Material
