# Supplementary material for: Minimalist Footwear Acutely Alters Running Kinematics in Runners With Medial Tibial Stress Syndrome
Source: Arthrosc Sports Med Rehabil. 2026 Apr 30;8(3):e70005. doi: 10.1002/ars2.70005 (PMC13307329; doi:10.1002/ars2.70005)
Supplement: Supplementary file 1 — Supplementary Material [file ARS2-8-e70005-s001.pdf]

SUPPORTING INFORMATION

APPENDIX I: Exploratory Subgroup Analysis Results

Table S1 and Table S2 presented kinematic changes and foot strike pattern shift specific for male and female subgroups when switching from standard to minimalist running shoes. Comparison between shoe condition for each subgroup was performed using the same statistical tests when comparing aggregated data: Wilcoxon signed-rank test for continuous variables and Stuart-Maxwell test for categorical variables.

TABLE S1

Average kinematic variables when wearing standard running shoes and those when wearing minimalist running shoes of each; average kinematics changes after transition to minimalist shoes. Data are presented for each sex subgroup as mean ± standard deviation. P-values were from Wilcoxon signed rank test comparing kinematic variables under two footwear conditions. *Significantly different with $p<0.05$; SRS = standard running shoes; MRS = minimalist running shoes.

**MALE DATA (N=19)**					
**Kinematic Variable**	**Laterality**	**SRS**	**MRS**	**Change**	**p-value**
**Peak Knee Flexion During Stance (°)**	Left	35.9 ± 10.8	33.6 ± 8.6	-2.2 ± 5.2	**0.008***
	Right	36.9 ± 9.2	33.5 ± 5.7	-3.4 ± 7.4	**0.024***
**Peak Hip Internal Rotation During Stance (°)**	Left	18.9 ± 15.4	19.8 ± 16.2	0.9 ± 5.9	0.872
	Right	21.3 ± 10.7	22.3 ± 10.7	1.0 ± 6.2	0.687
**Frontal Plane Pelvic Tilt Range (°)**	Left	5.6 ± 3.7	5.0 ± 2.0	-0.7 ± 4.2	0.809
	Right	5.6 ± 4.4	4.7 ± 2.3	-0.9 ± 3.3	0.334
**Stride Length (cm)**	-	193.9 ± 16.1	190.9 ± 15.0	-2.9 ± 3.6	**0.005***
**Cadence (step/min)**	-	172.8 ± 13.9	175.4 ± 14.1	2.6 ± 3.4	**0.008***
**FEMALE DATA (N=6)**					
**Kinematic Variable**	**Laterality**	**SRS**	**MRS**	**Change**	**p-value**
**Peak Knee Flexion During Stance (°)**	Left	43.1 ± 5.4	43.1 ± 5.9	0.0 ± 1.6	0.917
	Right	43.1 ± 3.5	42.3 ± 5.7	-0.7 ± 2.6	0.600
**Peak Hip Internal Rotation During Stance (°)**	Left	20.0 ± 7.3	14.3 ± 13.3	-5.7 ± 8.3	0.173
	Right	23.1 ± 6.6	16.6 ± 7.8	-6.6 ± 6.9	**0.046***

**Frontal Plane Pelvic Tilt Range (°)**	Left	4.5 ± 1.9	4.5 ± 2.1	0.0 ± 0.4	0.917
	Right	4.1 ± 1.9	4.3 ± 1.6	0.2 ± 1.0	0.600
**Stride Length (cm)**	-	185.2 ± 14.1	182.2 ± 12.6	-2.9 ± 3.9	0.116
**Cadence (step/min)**	-	180.9 ± 13.8	183.7 ± 12.6	2.8 ± 3.9	0.173

TABLE S2

Strike patterns frequency of both feet when running with different footwear time. Data were presented for each sex subgroup as frequency (percentage). P-values were from Stuart-Maxwell test comparing strike patterns between wearing standard and minimalist running shoes. SRS = standard running shoes; MRS = minimalist running shoes; FFS = forefoot strike; MFS = midfoot strike; RFS = rearfoot strike.

**Male Data (N=19)**				
**Foot Laterality**	**Strike Pattern**	**SRS**	**MRS**	**p-value**
**Left**	FFS	10 (53%)	7 (37%)	0.388
	MFS	6 (32%)	9 (47%)	
	RFS	3 (16%)	3 (16%)	
**Right**	FFS	9 (47%)	8 (42%)	0.779
	MFS	7 (37%)	7 (37%)	
	RFS	3 (16%)	4 (21%)	
**Female Data (N=6)**				
**Foot Laterality**	**Strike Pattern**	**SRS**	**MRS**	**p-value**
**Left**	FFS	3 (50%)	1 (17%)	0.157
	MFS	3 (50%)	5 (83%)	
	RFS	0 (0%)	0 (0%)	
**Right**	FFS	4 (67%)	1 (17%)	0.135
	MFS	1 (17%)	5 (83%)	
	RFS	1 (17%)	0 (0%)	
